# Current status of molecular diagnostic approaches using liquid biopsy

**DOI:** 10.1007/s00535-023-02024-4

**Published:** 2023-07-20

**Authors:** Kenji Takahashi, Yohei Takeda, Yusuke Ono, Hajime Isomoto, Yusuke Mizukami

**Affiliations:** 1 Division of Metabolism and Biosystemic Science, Gastroenterology, and Hematology/Oncology, Department of Medicine, Asahikawa Medical University, 2-1 Midorigaoka Higashi, Asahikawa, Hokkaido 078-8510 Japan; 2 Division of Medicine and Clinical Science, Department of Multidisciplinary Internal Medicine, Faculty of Medicine, Tottori University, Tottori, Japan; 3 Institute of Biomedical Research, Sapporo Higashi Tokushukai Hospital, Sapporo, Japan

**Keywords:** Pancreatic juice, Liquid biopsy, Pancreatic cancer, Gene mutation, Secretin

## Abstract

Pancreatic ductal adenocarcinoma (PDAC) is one of the most aggressive and lethal cancers, and developing an efficient and reliable approach for its early-stage diagnosis is urgently needed. Precancerous lesions of PDAC, such as pancreatic intraepithelial neoplasia (PanIN) and intraductal papillary mucinous neoplasms (IPMN), arise through multiple steps of driver gene alterations in *KRAS*, *TP53*, *CDKN2A*, *SMAD4*, or *GNAS*. Hallmark mutations play a role in tumor initiation and progression, and their detection in bodily fluids is crucial for diagnosis. Recently, liquid biopsy has gained attention as an approach to complement pathological diagnosis, and in addition to mutation signatures in cell-free DNA, cell-free RNA, and extracellular vesicles have been investigated as potential diagnostic and prognostic markers. Integrating such molecular information to revise the diagnostic criteria for pancreatic cancer can enable a better understanding of the pathogenesis underlying inter-patient heterogeneity, such as sensitivity to chemotherapy and disease outcomes. This review discusses the current diagnostic approaches and clinical applications of genetic analysis in pancreatic cancer and diagnostic attempts by liquid biopsy and molecular analyses using pancreatic juice, duodenal fluid, and blood samples. Emerging knowledge in the rapidly advancing liquid biopsy field is promising for molecular profiling and diagnosing pancreatic diseases with significant diversity.

## Introduction

Pancreatic ductal adenocarcinoma (PDAC) is the most dismal malignancy, with a 5-year survival rate of approximately 10% [1], as only < 25% of PDAC are localized and potentially curable during the first diagnostic phase [2, 3]. Owing to the lack of early-stage diagnosis, most patients with PDAC present with locally advanced or metastatic cancers [4]. Surgical resection that results in a better prognosis than other therapies [5] is an option; however, over 80% of PDAC cases are advanced owing to constant invasion and distant metastasis [6, 7]. FOLFIRINOX (fluorouracil, leucovorin, irinotecan, and oxaliplatin) and gemcitabine plus nanoparticle albumin-bound paclitaxel (GnP) are chemotherapeutic agents used to treat unresectable PDAC. However, their effectiveness is still unsatisfactory and limited [5].

For the early diagnosis of PDAC, imaging studies performed for screening or symptomatic examination should accurately detect pancreatic tumors, main pancreatic duct dilatation, and cystic changes [8]. Abdominal ultrasound is simple but does not have a high tumor detection rate; contrast agents have been reported to improve detection in recent years [9]. Computed tomography (CT) is the most widely used modality in diagnosing the presence and extent of PDAC [10]. However, to avoid radiation-associated malignancies in *BRCA* mutation carriers [11], follow-up is usually preferred using magnetic resonance imaging (MRI). To date, there is no clear evidence of a link between medical radiation exposure and accelerated pancreatic carcinogenesis; however, the long-term effects of frequent dynamic scanning need to be carefully evaluated.

Endoscopic ultrasonography (EUS) was developed in the early 1980s, and EUS-guided tissue acquisition (TA) was first reported in 1992 [12]. Since then, EUS has become increasingly widespread. EUS achieves the best detection performance [13]. However, it differs from other endoscopic procedures in its operation, making it challenging to train physicians and limiting its applicability as its diagnostic performance is influenced by the examiner's skill [14]. Attempts to strengthen cooperation among medical institutions are also being made to utilize these examination modalities accurately. [15, 16]. Significant efforts have been made to identify the etiology of carcinogenesis and its risk factors. However, strategies that integrate this knowledge into clinically feasible scoring are yet to be established [17]. Recently, scattered reports on using specific biomarkers for the minimally invasive identification of high-risk PDAC groups are available. However, to date, no effective biomarker for early detection has been established, despite an understanding of the mechanism of pancreatic carcinogenesis.

A liquid biopsy system using body fluids to detect reliable biomarkers is crucial for the early diagnosis of PDAC to improve diagnostic applications. Carbohydrate antigen 19–9 (CA19-9) and carcinoembryonic antigen (CEA) are commonly used tumor markers for PDAC. CA19-9 has the highest sensitivity at 70–80% and a < 50% specificity for PDAC diagnosis. Moreover, the sensitivity of CA19-9 for stage I PDAC is 55.6% [18]. Notably, several studies have reported the usefulness of CA19-9 as an anchor marker for PDAC [19, 20]; however, its utility is still restricted, particularly in Lewis antigen-negative patients characterized by inadequate secretion of CA19-9 and fucosylation deficiency [21].

In contrast, many clinical trials have been conducted to identify new biomarkers using liquid biopsy tools, such as cfDNA, cell-free RNA (cfRNA), proteins, extracellular vesicles (EVs) including exosomes, and circulating tumor cells (CTCs) [22, 23]. For example, the cell surface proteoglycan glypican-1 (GPC1) is present in PDAC cell-derived exosomes, and circulating exosome GPC1 in serum has been reported to serve as a biomarker for detecting early stages of PDAC [24]. Moreover, several miRNAs or exosomal miRNAs are highly expressed in body fluids such as blood, duodenal fluid (DF), and pancreatic juice (PJ) [25–27]. We previously encountered a case of early-stage PDAC in which *KRAS* mutations were detected in PJ within the resected PDAC tissue but not in the plasma, suggesting that PJ is superior to blood for PDAC diagnosis as it may reflect DNA mutations or abnormal RNA expression from the original tumor more precisely owing to the short distance between the primary lesion and the position from where PJ is collected [28]. DF can also be a valuable sample collected using a minimally invasive method compared with PJ collection using a catheter. Nevertheless, there is a need to discover and validate new markers in PJ and DF for their utility for liquid biopsy to enable the early diagnosis of PDAC and increase the opportunities for curative surgery.

This review summarizes the gene alterations associated with initiating PDAC precursor lesions, such as intraepithelial neoplasia (PanIN) and intraductal papillary mucinous neoplasms (IPMN). It discusses the molecular subtypes of invasive PDAC. We also discuss literature and trials on the early diagnosis of PDAC. Finally, we summarize the key themes and recent progress in liquid biopsy using body fluids, mainly PJ and DF, to analyze oncogenic mutations, RNA expression, and other factors, focusing on high-grade IPMN and PDAC.

## Precancerous lesions and gene alterations

### Pancreatic intraepithelial neoplasia (PanIN) and gene alteration

**Fig. 1:**
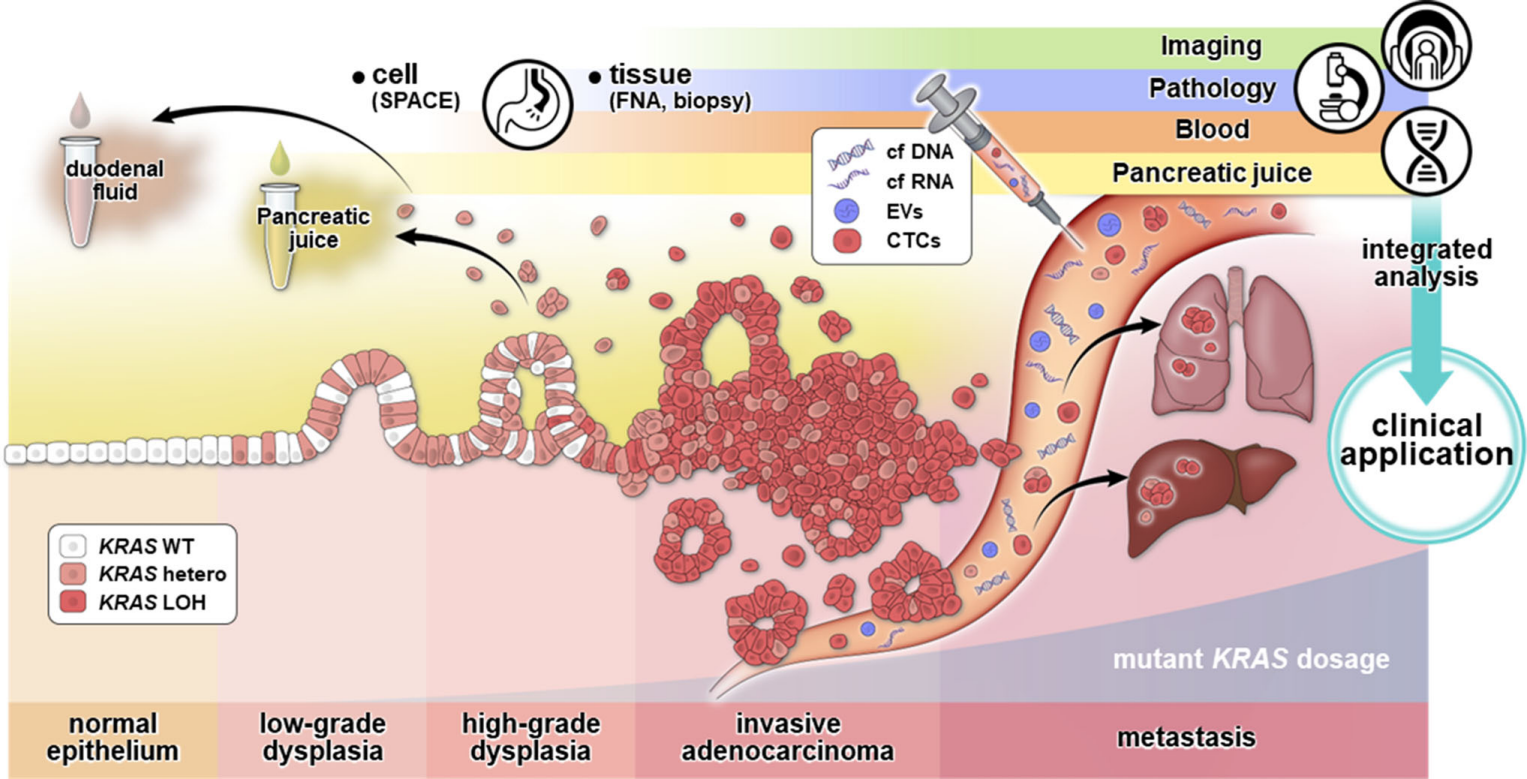
Diagnostic material/information during pancreatic carcinogenesis. Oncogenic *KRAS* can initiate precursor lesions (dysplasia; see Fig. 2 for details), and allelic imbalance increases oncogenic dosage gain and tumor progression. In addition to circulating tumor cells (CTCs), mutations and methylations in circulating cell-free DNA (cfDNA), cell-free RNA (cfRNA), and extracellular vesicles (EVs) can serve as liquid biopsy targets. A multi-layer detection system covering these factors is necessary for early diagnosis, complementary to conventional pathological assessments (e.g., SPACE and FNA). In addition to blood, where tumor-derived factors are significantly diluted, the collection of DF and PJ is expected to provide an opportunity to capture such molecules. Such genomic information can be integrated with traditional diagnostic modalities, improving the accuracy of early detection of human pancreatic cancer with high accuracy

PanIN is the most common precursor lesion for PDAC. Oncogenic *Kras* can induce PanIN in mice [29], and humans with PDAC harbor *KRAS* mutations. Tumor development is initiated by KRAS mutation at the earliest stages of pancreatic tumorigenesis [30]. Mutations are mainly detected in codon 12, while during the initial stage of pancreatic carcinogenesis, mutations are occasionally identified in codons 13 and 61 [31]. Approximately 70–80% of PDAC patients harbor G12V, G12D, or G12R *KRAS* mutations [32, 33]. *KRAS* G12D mutation is the most frequent and is a predictive factor for worse PDAC prognosis [34]. A metastatic mouse PDAC model with Kras^G12D^ revealed that Kras mutant allele-specific imbalances, such as copy number gain or loss of heterozygosity, were observed even in human PanIN lesions and invasive PDAC (Fig. 1) [35].

Following tumor initiation by *KRAS* mutation, PanIN lesions progress to invasive PDAC through subsequent inactivation of the tumor suppressor genes *TP53*, cyclin-dependent kinase inhibitor 2A (*CDKN2A*), and SMAD family member 4 (*SMAD4*) [36]. Mutations in several genes have been associated with PDAC; however, these four genes are the primary driver genes for human PDAC [37].

### IPMN and gene alteration

IPMN has been associated with *GNAS* mutation at codon 201 in 40–70%, while *KRAS* mutations in 40–65% of cases [36]. *GNAS* gene is located on chromosome 20q13.32 and encodes the G-protein alpha-subunit [G(s)α] of heterotrimeric G-protein-coupled receptors. Activation of protein kinase A (PKA) through stimulation of the messenger cAMP by the ligand induces the phosphorylation of cAMP-responsive element-binding protein (CREB) and extracellular signal-regulated kinase (ERK). In several tissues, GNAS–cAMP signaling maintains cellular differentiation and quiescence [38–41]. *GNAS* mutations have been detected in most intestinal-type and about 50% of gastric-type IPMNs [42]. *GNAS* mutations can evolve from gastric epithelial-type to intestinal-type IPMNs via the induction of intrinsic CDX2/MUC2 expression [43]. *GNAS* mutations are crucial for IPMN initiation [44]. However, they have also been shown to suppress tumor development in several types of cancers, including basal cell carcinoma and medulloblastoma [45, 46]. We recently reported that *GNAS* mutations inhibit tumor cell invasion by suppressing the KRAS pathway in PDAC [39]. These results suggest that GNAS may have inconsistent roles with tumor-initiating and tumor-suppressing effects.

Germline mutations in Serine/Threonine Kinase 11 (STK11) are considered a major cause of Peutz-Jeghers syndrome [47]. A recent study demonstrated that consistent loss or reduction of STK11 expression was observed in 14% of IPMNs, and the aberrant tumor suppressor proteins were associated with *STK11* mutation in 58% of IPMNs and significantly downregulated phosphorylated AMPK levels [48]. In this subset, *KRAS* mutations were observed in 92% of cases, whereas *GNAS* mutations were detected in none. *STK11* mutations are frequently observed in pancreatobiliary types of IPMN, and patients with aberrant STK11 have poorer survival than those with normal STK11.

*RNF43* is a tumor suppressor gene, and loss-of-function mutations in *RNF43* often accompany *GNAS* mutations during IPMN development [49]. RNF43 serves as a negative feedback regulator of Wnt signaling by suppressing the membrane expression of Frizzled, and *RNF43* mutation confers Wnt dependency on PDAC cells [50]. In addition, this gene is involved in the ATM–ATR-mediated DNA damage response pathway, and its loss promotes tumor development by bypassing oncogene-induced senescence [51]. An analysis of resected IPMN tissue and mucinous cystic neoplasm (MCN) revealed *RNF43* mutations in 56% of cases, which were more frequent in non-invasive lesions than invasive lesions [52]. Similarly, Kruppel-like factor 4 (*KLF4*) mutation was detected in over 50% of resected IPMN and significantly more prevalent in low-grade IPMN than in high-grade IPMN [53]. Considering the increased occurrence of *RNF43* and *KLF4* mutations in low-grade tumors, alterations in these genes likely have less impact on clonal expansion during IPMN progression. In contrast, TP53, CDKN2A, and SMAD4 mutations are more common with high-grade dysplasia (HGD), supporting their role in risk stratification for IPMN progression to invasive tumors [52, 54].

### Approaches for defining molecular subtypes of PDAC and their utility in early diagnosis

The cell origin theory is crucial to understand better the diversity of clonal evolution in cancer [55]. Studies in genetically engineered mouse models (GEMMs) suggest that PanIN arises from pancreatic acinar cells that incur *Kras* mutations and undergo acinar-ductal metaplasia (ADM), characterized by the transformation of acinar cells into duct-like cells expressing CK19 and Sox9 [56, 57]. However, whether human PDA also undergoes ADM remains to be determined [58]. A recent study by Huang et al. proposed a potential cell of origin for PanIN or IPMN using human pluripotent stem cell-derived pancreatic progenitors and organoids [59]. Duct-specific expression of *GNAS* mutation is sufficient to induce IPMN lesions, and *GNAS* R201C causes cystic growth more effectively in the ducts than in the acinar organoids. In contrast, *KRAS* G12D induces cancerous lesions more often in acinar versus ductal organoids [59]. These data suggest that PanIN and IPMN may originate from acinar and ductal cells.

Transcriptomic subtyping approaches have been developed over the past 10 years to understand better the pathology of PDAC, evolving a framework for molecular taxonomy [60]. PDAC can be classified into two common molecular subtypes: basal like and classical. The basal-like subtype is characterized by a more aggressive phenotype and poor patient survival, whereas the classical subtype is highly sensitive to chemotherapeutic agents. Therefore, knowing which subtype is involved can help guide chemotherapy decisions [61–63]. Phenotype classification by RNA signatures may not be clinically relevant because of the high cost and difficulty in analyzing heterogeneous tumors. However, immunohistochemical analysis has several advantages in overcoming this issue; GATA6, CK5, and Vimentin may serve as relevant markers for defining the differential expression profile of heterogeneous tumors [64]. Like GATA6, GATA4 maintains the classical phenotype in cooperation with GATA6 [65].

**Fig. 2:**
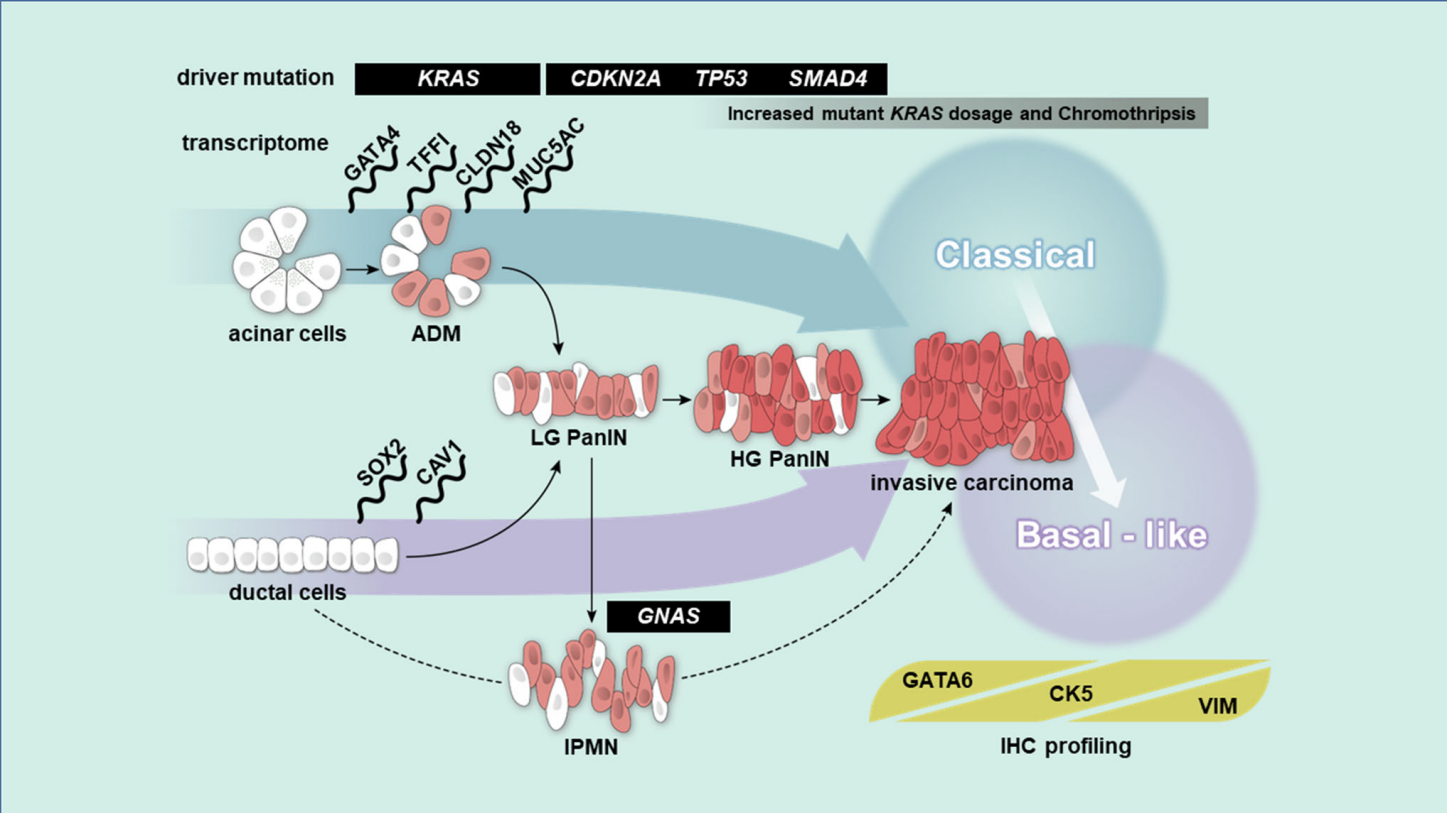
Cell of origin and molecular subtypes in pancreatic cancer. The pancreas contains several specialized cell types that can alter cellular identity in response to inflammation and subsequent regeneration/repair. Pancreatic acinar cells can undergo acinar-to-ductal metaplasia (ADM), a reprogramming event that induces transdifferentiation into a duct-like phenotype. Pancreatic intraepithelial neoplasia (PanIN)-like lesions can be generated in mice through the expression of oncogenic *Kras* in acinar cells; intraductal papillary mucinous neoplasms (IPMN) can arise from acinar cells in association with mutant *Kras* and *Gnas* or from ductal cells in the context of association with chromatin remodeling or transforming growth factor β signaling mutations. The subsequent inactivation of tumor suppressors accelerates the progression of precursor lesions, leading to invasive ductal adenocarcinoma. Molecular subtyping approaches for pancreatic cancer based on transcriptomic data can help stratify patients in clinical practice, leading to optimized treatment algorithms. The recently identified classical and basal-like molecular subtypes of pancreatic cancer affect patient survival and can be profiled by the differential immunohistochemical expression of GATA6, CK5, and Vimentin. Phenotypic transitions can be observed during chemotherapy and even during the natural history of tumor progression via genetic and epigenetic alterations. It remains to be determined if mutant *KRAS* dosage and chromothripsis regulate PDAC development. Recent mouse and human studies have supported the significant role of the cell of origin and the associated transcriptomes in influencing PDAC phenotype

Flowers et al. suggested that ductal cell-derived tumor signatures are associated with the basal-like subtype. However, acinar cell-derived tumor signatures are correlated with the classical subtype of human PDAC, using a gene set from GEMM in the context of oncogenic *Kras* and *Tp53*. They also suggested that specific genetic events, such as mutations in *TP53* and *KDM6A*, may be associated with particular subtypes [66]. There is some confusion, however, regarding IPMN-related tumors. As IPMN likely originates from ductal cells, this would suggest that it is correlated with the more aggressive basal-like subtype of PDAC. Yet Collisson et al. have identified IPMN-related tumors as belonging to the classical subtype, which Flowers et al. associate with acinar cells related to PanIN [60]. This raises a discrepancy between these two theories: cell of origin and molecular subtypes (Fig. 2). Moreover, in IPMN, driver gene mutations, such as *GNAS, STK11, RNF43,* or *KLF4*, may also determine the molecular subtype and its transition [32, 67]. Therefore, the regulation of PDAC subtypes and their potential interaction with cancer cell origin must be further investigated in early-stage tumors to develop novel clinical applications for screening and surveillance.

Body fluids transcriptomes can provide clinically relevant information to explore the origin of cancer and the roots of PDAC subtypes. Larson et al. reported that plasma cell-free RNAs (cfRNAs) in plasma help detect cancer tissue origin and subtypes [68]. The circulating cell-free genome atlas (NCT02889978) contains transcriptome data of blood samples from cancer and non-cancer patients. To date, no registry has collected mutation profiles combined with transcriptome analysis using DF or PJ, and we are currently investigating the states of driver mutations and transcriptomes in DF and PJ in collaboration with affiliated hospitals to establish a catalog for identifying PDAC subtypes and detecting the early stages of tumors more effectively than blood testing.

## A diagnostic approach for early-stage PDAC

### Endoscopic modalities for pathological assessment

The pathological diagnosis of PDAC is mandatory for patients with unresectable PDAC, and its significance has increased with advances in matched therapies following molecular profiling [69]. There has been an increase in the use of preoperative chemotherapy and a growing consensus that pathological diagnosis is crucial in cases where surgery is an option [70, 71].

EUS-TA, with a sensitivity of 84–91%, is the most widely performed tissue sampling method for pathological diagnosis [72, 73]. EUS-TA has progressed with a focus on puncture needles, such as forward facing, Franseen, and fork tips [74–77], and specimen collection and processing methods, such as rapid on-site evaluation and target sample check illuminator [78, 79]. Tissues obtained by EUS-FNA are occasionally small, and a diagnosis may not be made in samples containing a large amount of blood. Some reports have suggested that increased punctures improve diagnostic performance [80].

An unusual complication associated with EUS-TA is needle tract seeding. Kitano et al. reported that transgastric but not transduodenal EUS-TA causes seeding in approximately 0.9% of cases [81]. Therefore, careful monitoring is required following the resection of PDACs diagnosed using EUS-TA. In addition, the procedure should be avoided in cases where it is difficult to suspend antithrombotic drugs, where it is challenging to delineate the mass by EUS, or where the presence of a blood vessel or pancreatic duct in the puncture route is unavoidable.

Pancreatic juice cytology (PJC), with a sensitivity of 47–76%, is an alternative to EUS-TA [82]. Notably, several methods, including brushing PJC, washing PJC, endoscopic nasopancreatic drainage (ENPD) with serial pancreatic juice aspiration cytologic examination (SPACE) [83], and secretin-loaded PJC (S-PJC), have been attempted to improve the diagnostic performance of PJC [84]. However, all methods are associated with the risk of post-ERCP pancreatitis as a severe complication and are still considered alternatives to EUS-TA. Iiboshi et al. reported the usefulness of multiple consecutive PJC examinations with ENPD [83]. Sensitivity, specificity, and overall accuracy were 100%, 83.3%, and 95%, respectively, with an average of 5.3 mL PJ sampling. They also reported that SPACE is particularly useful for diagnosing intraepithelial carcinomas that cannot be delineated using EUS. Since then, there have been increasing reports on diagnosing intraepithelial pancreatic cancer using SPACE, mainly in Japan [85–88].

Until recently, S-PJC had been performed widely using porcine secretin [84, 89]; however, animal-derived products have been discontinued due to safety issues. Synthetic human secretin is now available as a secretin preparation. In the United States, synthetic secretin is licensed for use in (i) the exocrine pancreas function test, (ii) the Zollinger-Ellison Syndrome Test, and (iii) stimulation of pancreatic secretions to facilitate the identification of the ampulla of Vater and accessory papilla during ERCP. However, secretin has not been approved for clinical use in some countries, including Japan. The unstable supply of secretin is another issue. Mass production, distribution, and cost reductions are expected as its clinical utility becomes clearer. Other methods include (1) bile cytology in cases of pancreatic head cancer invading the distal bile duct [89], (2) ascites cytology in cases of peritoneal dissemination with exudative ascites effusion, and (3) tumor biopsy under transabdominal ultrasound or CT in institutions where EUS-TA is not feasible [90].

### Clinical application of genetic testing

A highly sensitive method for detecting even minor changes in crucial genes is required, particularly in the early stages of cancer. Invariably, numerous techniques have been developed to quantify mutations in driver oncogenes and tumor suppressor genes. One method fundamental in mutation analyses is polymerase chain reaction (PCR). Real-time PCR for mutation-specific amplification has been widely validated with high sensitivity and reliability, allowing for the real-time detection and quantification of DNA in a comparatively short turnaround time.

Further technological developments aimed at improving detection sensitivity have led to the advent of technologies such as the BEAMing (Beads, Emulsion, Amplification, and Magnetics) method [91] and digital PCR (dPCR) [92]. dPCR is a highly sensitive method that performs endpoint PCR on more than 10,000 individual reaction wells (such as oil droplets or chambers), allowing for accurate detection and absolute quantification of DNA mutations without calibration reactions [93]. The dPCR assays achieved a shallow variant allele frequency (VAF) detection limit (approximately 20-fold; 0.05% vs. 1%). The multiplex droplet digital PCR assay was developed to target many genomic regions using a few primers and probes [94–96]. As tissue specimens are occasionally minimal, comprehensive analysis of genomic alterations in a single reaction is a powerful tool for clinical testing, potentially compensating for pathological diagnosis. The multiplex ddPCR assay can detect point mutations in cfDNA to minimize false positives while retaining sensitivity, making it feasible to analyze cfDNA samples [97].

Comprehensive genomic profiling (CGP) using next-generation sequencing (NGS) is the most powerful tool for identifying tumor-specific genomic alterations in clinical oncology, enabling precision medicine [98–100]. In addition to clinically available CGP assays such as FoundationOne CDx and OncoGuide, whole exome sequencing (WES) and whole genome sequencing (WGS) are used to evaluate to fine-tune cancer therapy for patients with unresectable diseases [101]. As a super-sensitive analysis protocol for cfDNA, unique molecular identifiers (UMIs) that are attached to unique DNA sequences in each original molecule of DNA are standard tools for overcoming PCR and sequencing errors to reliably quantitate low-frequent mutations at ≤ 0.1% VAFs [99, 100]. In addition to determining the best cancer therapy, the possibility of early PDAC screening through cfDNA diagnostics has been widely discussed, and many trials on cfDNA mutation profiling have been conducted [102]. Such a technique would be useful in stratifying high-risk individuals, such as those with a family history of PDA and pancreatic cysts, including IPMN. Furthermore, several other CGP panels for tissue and plasma cfDNA using genetic or epigenetic biomarkers, such as FoundationOne Liquid [103] and Guardant360, as well as the more recent CGP: GuardantOMNI and GuardantINFINITY [104], have also been developed. Currently, the last two panels are even more comprehensive for research use only and may eventually allow diagnosis at an earlier tumor stage [105].

## Molecular diagnosis using body fluids

### Advantage of the analysis using PJ or DF

Molecular diagnostic methods using body fluids have been developed in recent years, and we outlined the benefits and the challenges that need to be addressed in Table 1. Significant efforts have been made to detect mutations in plasma cfDNA in many cancer types early. Such detection may be a biomarker of tumor burden; however, mutation detection rates are lower in patients with PDAC than in those with other neoplasms, primarily because of low tumor cellularity [106]. Even in metastatic PDAC, a previous study demonstrated that the accuracy only reached around 40% [107]. Considering the low yield of circulating plasma cfDNA, specifically in patients with small PDAC, detecting tumor-derived DNA remains challenging [108]. Therefore, alternative approaches must be considered to overcome this limitation.

**Table 1:** The benefits and current challenges of each molecular diagnostic method for PDAC

Sample	Analysis target	Molecular targets	Features and benefits	Current problems and challenges
Body fluid (blood, DF, PJ, etc.)	Cell-free DNA (cfDNA)	*KRAS, TP53, SMAD4, CDKN2A, GNAS, RNF43,* etc.	Plasma cfDNA concentration in 1–10 ng/mL and is increased in patients with cancers Analysis of cfDNA is a well-developed technique cfDNA and cfRNA represent complete tumor heterogeneity They are useful for monitoring treatment response, development of resistance, and tumor recurrence	Clinical practice rules are not well established Rapidly degrade in body fluid samples Difficult to detect the origin of a tumor Experience potential contamination with DNA and RNA from normal cells
	Cell-free RNA (cfRNA)	*miR-21, miR-10b, miR-30c, miR-181a, miR-17, miR-2346, HULC, HOTAIR, LINC00346, ABHD11-AS1,* etc.		
	Extracellular vesicles (EVs)	*miR-17, miR-21, miR-23a, miR-34a, miR-106a, miR-108a, miR-423, miR-1246, miR-4634,* CD63, CD9, CD81, *HULC, Sox2ot, UCA1, MALAT1, CRNDE,* MUC 5AC, glypican-1, etc.	EVs contain RNA, protein, and other tumor cell-derived factors EVs are found in most body fluids	Analysis methods are diverse and still under development
	Circulating tumor cells (CTCs)	EpCAM, cytokeratin, MUC1, etc.	CTC contents (DNA, RNA, and proteins) are examined as liquid biopsy markers CTCs have broad utility Morphological and functional analyses are possible	Extreme rarity, fragility, and heterogeneity of CTCs Analysis methods are diverse and still under development
Tumor tissue (biopsy/cytology)	DNA, RNA, Protein, etc.	*KRAS, TP53, SMAD4, CDKN2A, GNAS, RNF43, STK11,* etc.	Clinically validated methods	Invasive to patients Fail to show tumor heterogeneity and distant metastasis Difficult for monitoring therapeutic effect and tumor recurrence

Analysis of blood cfDNA from patients with pancreatic neoplasms has been performed using high-sensitivity dPCR technology [109]. dPCR detects *KRAS* codon12 mutations even in pancreatic cancer cohorts with many early-stage patients [110]. We developed a dPCR method to overcome subsampling errors, an issue in testing for extremely low copy number mutations [111]. Using the pre-amplification method, tumor-derived mutant *KRAS* in the plasma of patients with resectable PDAC was accurately detected (AUC, 0.861–0.876), and the dPCR method improved post-resection recurrence prediction over that of the marker CA19-9 [26]. DNA in blood exosomes (exoDNA) is protected from degradation and fragmentation and can be extracted as high molecular weight DNA compared with cfDNA. It has been reported that exoDNA levels increase after neoadjuvant therapy; exoDNA also yields a higher detection rate of *KRAS* mutations with dPCR and is more amenable as a template for CGP [112].

In gastrointestinal organs, secreted digestive juices may enrich genetic abnormalities associated with cancerous lesions. Therefore, liquid biopsy using body fluids could resolve the limitations of mutation detection in plasma cfDNA. Initially, *KRAS* mutations were targeted using PJ collected from patients with PDAC/IPMN for PJC and could complement the less-sensitive cytology evaluation and help in follow-up [113–115]. Using high-resolution melt curve analysis and pyrosequencing methods [31], mutations in *KRAS* are detected in PJ from PDAC patients and in 50% of asymptomatic individuals at high risk for neoplasms [116]. *KRAS* mutations have also been observed in individuals without pancreatic abnormalities, likely because of invisible PanIN lesions. *TP53* mutations have also been detected in PJ from patients with HGD [31].

Sequencing technologies enable comprehensive cancer genome profiling using PJ and efficient detection of tumor suppressor mutations, including *TP53, KRAS,* and *GNAS* mutations, serving as a tool to stratify tumor grades in patients with IPMN [117, 118]. Yu et al. developed a digital NGS method in which many aliquots of DNA from each patient’s fluid were individually subjected to sequencing, and a mutation score of one was given to each mutation-containing aliquot [119]. Canonical mutations during pancreatic carcinogenesis (e.g., *KRAS, TP53, SMAD4,* and *CDKN2A*), using this method, were efficiently detected in the PJ of PDAC/IPMN patients, proving useful in the evaluation of patients undergoing pancreatic surveillance [120].

Epigenetics, fragmentomics, and cfDNA topology are crucial for developing DNA-based cancer screening [121]. Advanced library preparation was adapted to allow for the methylome profiling of samples with a low input of DNA (1–10 ng), potentially applicable to early cancer detection [122]. In addition to this technology, detailed informatics regarding pan-cancer methylome analysis offer a better classification of cancer origins [123]. Methylated DNA in the PJ can also provide clinically relevant information to diagnose PDAC, and three methylated DNA markers (C13orf18, FER1L4, and BMP3) can distinguish non-PDAC patients from PDAC patients, including those in the early stage or those with HG IPMN [124].

Recently, cfRNAs and EVs were investigated as potential tools for liquid biopsy (Table 2) [23, 125]. EVs comprise exosomes, apoptotic bodies, and microvesicles and are released by most cells, including cancer cells. EVs are microstructures with a lipid bilayer membrane containing RNAs, proteins, or lipids and transfer their contents from donor to recipient cells [126–128]. As EVs exist in circulating body fluids, such as blood, PJ, and DF, the expression levels of their contents, mRNA, protein, and non-coding RNAs (ncRNAs), have been used as biomarkers for PDAC diagnosis [25]. ncRNAs are non-protein-coding RNAs that regulate diverse biological processes in several diseases [129]. In particular, ncRNAs, non-protein-coding RNAs regulating various biological processes in several diseases, have recently been investigated regarding disease pathogenesis, including several cancers. They are divided into two main groups based on their transcript size. Short ncRNAs, which are less than 200 nucleotides in length, include microRNAs (miRNAs) and other small RNA classes, whereas long non-coding RNAs (lncRNAs) are greater than 200 nucleotides long [129–131].

**Table 2:** Examinations of body fluid-derived EVs or RNAs as liquid biopsy tools for PDAC

Target	Type of clinical sample	Potential roles for liquid biopsy for PDAC diagnosis	References
miR-21	Serum EVs	EVs miR-21 was upregulated in patients with PDAC compared to healthy individuals and be identified as a prognostic and chemo-resistant marker	Goto et al. [132]
miR-1246, miR-4644, miR-3976, and miR-4306, CD44, EpCAM	Serum EVs	The combined analysis of serum EVs miRNAs with CD44 and EpCAM could contribute to detecting the early stages of PDAC	Madhavan et al. [133]
MUC5AC	Plasma EVs	MUC5AC expression showed significantly higher levels in invasive IPMN compared with low-grade IPMN	Yang et al. [134]
MALAT-1	Serum EVs	Serum EVs MALAT-1 was increased in PDAC patients compared to IPMN patients or healthy individuals	Kumar et al. [135]
HOTAIR	Serum	HOTAIR in serum was increased in PDAC patients compared to healthy individuals	Ma et al. [136]
Sox2ot	Plasma EVs	Plasma EVs Sox2ot was highly expressed in PDAC patients and associated with TNM stage	Li et al. [137]
HULC	Serum EVs	EVs HULC in serum was highly expressed in serum from PDAC patients compared to IPMN patients or healthy individuals	Takahashi et al. [144]
miR-21, miR-155	PJ EVs	MiR-21 and miR-155 within exosomes discriminated PDAC patients compared with chronic pancreatitis (CP) patients	Nakamura et al. [27]
miR-21, miR-25, miR-16	PJ EVs	The combination of EV miR-21, miR-25, and miR-16 with serum miR-210 and CA-19–9 had an AUC of 0.91, a specificity of 84.2%, and a sensitivity of 81.5% for PDAC diagnosis	Nesteruk et al. [138]
Size and concentration of EVs	PJ EVs	EVs concentration did not differ between healthy control and PDAC patients. PJ from PDAC had a higher number of large vesicles	Nesteruk et al. [138]
MUC1, MUC4, MUC5AC, MUC6, MUC16, CFTR, MDR1	PJ EVs	MUC1, MUC4, MUC5AC, MUC6, MUC16, CFTR, and MDR1 were increased in pancreatic juice-derived EVs of PDAC patients	Osteikoetxea et al. [139]
CEACAM 1/5, tenascin C	PJ EVs	Exosomal proteins, CEACAM 1/5 and tenascin C, were identified to be the most discriminating proteins between PDAC patients and benign controls	Zheng et al. [140]

EVs, extracellular vesicles; PDAC, pancreatic ductal adenocarcinoma; PJ, pancreatic juice;

In a blood examination, serum EV-encapsulated miR-21 was upregulated in patients with PDAC compared with healthy individuals and has been identified as a prognostic and chemoresistance marker [132]. Combined analysis of serum EVs miRNAs, miR-1246, miR-4644, miR-3976, and miR-4306, with PDAC-initiating cell markers, such as CD44 and EpCAM, could contribute to the detection of the early stages of PDAC [133]. Yang et al. investigated the usefulness of the expression levels of 16 plasma EV-related proteins as biomarkers of the malignant potential of IPMN. They found that MUC5AC expression was significantly higher in IPMN-associated carcinoma than in low-grade IPMN [134]. In contrast, some lncRNAs are detected as cfRNAs or EV-encapsulated RNAs in blood [25]. lncRNA MALAT-1 and HOTAIR expression is significantly increased in blood derived from patients with PDAC, suggesting their utility as biomarkers [141, 142]. Furthermore, lncRNA Sox2ot is present in plasma EVs and is highly expressed in patients with PDAC [137].

Nakamura et al. reported that expression levels of miR-21 and miR-155 within exosomes distinguished PDAC patients from chronic pancreatitis (CP) patients [27]; however, PDAC and CP patients could not be characterized based on miR-21 and miR-155 levels when whole RNAs from PJ were analyzed. The accuracy of exosomal miR-21 and miR-155 levels and PJC was 83%, 89%, and 74%, respectively. Another study revealed that EV-derived miR-21, miR-25, and miR-16 in the PJ were increased in patients with PDAC than in non-malignant controls. Combining these EV miRNAs with serum miR-210 and CA-19-9 showed an area under the curve of 0.91, a specificity of 84.2%, and a sensitivity of 81.5% for PDAC diagnosis [138]. They also investigated the size and concentration of EVs in PJ following secretin stimulation. EV concentrations did not differ between healthy controls and patients with PDAC; however, the PJ from PDAC had more large vesicles [143]. Moreover, in PJ-derived EVs, MUC1, MUC4, MUC5AC, MUC6, MUC16, CFTR, and MDR1 have been recognized as candidate markers for PDAC [139]. In addition, exosomal proteins carcinoembryonic antigen-related cell adhesion molecule (CEACAM) 1/5 and tenascin C have been identified as the most discriminating proteins between patients with PDAC and benign controls [140].

Besides miRNAs, mRNAs, and proteins, EV-encapsulated lncRNAs in PJ are yet to be investigated. We previously reported that EV lncRNA HULC in the serum was highly expressed in PDAC patients compared with IPMN patients or healthy individuals and served as a potential biomarker [144]. Therefore, similar to blood samples, several lncRNAs in the PJ may be crucial markers for detecting the early stages of PDAC. Moreover, no studies have evaluated EVs or cfRNAs to diagnose PDAC using DF. Considering the evidence showing that ncRNAs in the blood can be assessed through liquid biopsy [25], the analysis of ncRNAs and EVs is needed to discover previously unrecognized biomarkers in body fluids such as PJ and DF.

The relationship between the microbiome and PDAC has been increasingly reported in the past decade. In 2015, Fusobacterium species were found in PDAC tumors [145]. In 2019, Malassezia, a type of fungus, was shown to be increased in the pancreas of PDAC patients by 18S rRNA sequencing [146]. In 2022, certain strains of gut microbiota were shown to be more prevalent in patients with pancreatic cancer across racial groups (Japanese, Spanish, and German) using shotgun metagenomic analysis [147]. Examination of the PJ microbiome in resected pancreatic cancer tissue samples revealed no specific trends [148]. There remains much potential for microbiome research, not only as a direct cause of pancreatic carcinogenesis but also for the search for biomarkers for early diagnosis, the identification of new risk factors, and the prediction of treatment efficacy after diagnosis.

In this review, we have examined various studies conducted in different laboratories. However, it is difficult to determine the levels of evidence in each study. To address this issue, we need to establish specific protocols for liquid biopsies that standardize the sample collection process and ensure quality control throughout the molecular analysis process. By doing so, we can improve the consistency between investigators and better identify the origin tumor using liquid biopsy. In addition, integrating the information on where and how liquid biopsy factors are released into the body fluid and collected, sample size, single institute or multicenter study, and prospective study or study using existing samples will enhance compatibility across studies and deepen our understanding of the topics.

### Ongoing projects using PJ or DF

We have been studying the PJC using synthetic secretin since 2012 and are conducting a multicenter study on MRI imaging and liquid biopsy in addition to PJC (CRB6200003). Synthetic secretins such as ChiRhoStim (ChiRho-Clin, Inc., Burtonsville, MD, USA) and Secrelux (Sanochemia, Vienna, Austria) have been used in the past; the former is currently in use. We previously reported that administering 0.6 μg of synthetic human secretin increases the amount of PJ collected using a catheter-inserted transpapillary [149, 150]. For the substantial masses, collected volume increased from 2.0 ± 2.1 mL (range, 0–14.0 mL) to 3.7 ± 2.5 mL (range, 1–15.0 mL) in PDAC patients [149] and from 3.7 ± 7.3 mL (range:0–79.0 mL) to 5.1 ± 6.2 mL (range:1–64.0 mL) in IPMN patients [150]. Furthermore, in diagnosing malignancy using PJC, we found that the sensitivity improved from 50.9 to 74.0% and from 50.0 to 70.8% in the case of a substantial mass and IPMN, respectively [149, 150].

We also evaluated mutation detection in DNA from DF (DFDNA) as an alternative to plasma DNA (UMIN000028284). In 150 patients suspected of PDAC and IPMN, DF containing PJ was collected from the duodenal lumen through an endoscopic channel. DFDNA yielded significantly higher levels of plasma cfDNA and exhibited characteristic fragmentation patterns. Using molecular barcode sequencing, hotspot mutations detected in DF showed higher concordance with resected tissue than with plasma cfDNA. In contrast to plasma cfDNA, in which liquid tissue matches were limited to advanced-stage cancer, the DFDNA assay enabled higher concordance across a wide range of PDA stages. We showed that DF-derived DNA could be a potent biospecimen for detecting mutations with higher yield and sensitivity than plasma cfDNA [120].

In contrast, genuine PJ-derived DNA had a much higher mutation detection rate. During endoscopic retrograde cholangiopancreatography (ERCP) testing, pancreatic fluid was aspirated via a catheter from the main pancreatic duct using a contrast agent. Mutation analysis using molecular barcoding of DNA from 1 mL of PJ showed a much higher concordance rate for PJDNA than for DFDNA. Furthermore, in addition to mutations derived from the index lesion, several other mutations that might have originated from multicentric lesions in the residual pancreas were detected in the PJDNA (manuscript in preparation) [151]. Therefore, mutational analysis using PJDNA may be helpful for auxiliary diagnosis when PJ is collected for cytological analysis.

### Future directions

To investigate the usefulness of PJ-derived genes or EVs, we have initiated a prospective multicenter study to analyze EV RNAs, including lncRNAs and cfDNAs, in the PJ and plasma as liquid biopsy tools for the early diagnosis of PDAC (CRB6200003). Based on our previous report demonstrating the efficacy of synthetic secretin injections on the diagnostic accuracy of PJC [149], PJ samples are now collected following secretin administration. The dataset of DNA mutations and EV RNA expression in plasma and PJ will be integrated to develop a multi-layer diagnostic agonism for the early stages of PDAC diagnosis. These trials may offer new insights for developing diagnostic strategies and identifying therapeutic targets for deadly cancers.

## Perspectives

The utility of liquid biopsy is increasingly being explored, and several markers for the early diagnosis of human PDAC in humans are being identified. Novel cancer-associated RNAs as biomarkers and their roles in regulating tumor phenotypes are being investigated. In particular, ncRNAs seem promising for defining disease pathogenesis and serving as diagnostic and prognostic markers and therapeutic targets. Mutation analysis of liquid samples can supplement pathological diagnosis or may even be an alternative; however, there is a need to improve the diagnostic accuracy and identification of early PDAC using body fluids such as blood and PJ, and DF obtained via endoscopy. Therefore, a clinical application system must be promptly developed based on current and future trials. In addition, focused studies on the genes implicated in pancreatic diseases must be conducted to improve our understanding of disease pathogenesis and to aid their utility as diagnostic markers, so they can eventually lead to novel clinical applications.
